# Thyroid nodules and thyroid cancer in women with positive thyroid screening in pregnancy: a double-centric, retrospective, cohort study

**DOI:** 10.1530/ETJ-21-0011

**Published:** 2022-02-02

**Authors:** Jan Jiskra, Jiří Horáček, Sylvie Špitálníková, Jan Paleček, Zdeňka Límanová, Jan Krátký, Drahomíra Springer, Kristýna Žabková, Hana Vítková

**Affiliations:** 1 3rd Department of Medicine, 1st Faculty of Medicine, Charles University, General University Hospital, Prague, Czech Republic; 2 4th Department of Medicine, Faculty of Medicine, Charles University, University Hospital Hradec Králové, Czech Republic; 3 Department of Nuclear Medicine, District Hospital, Havlíčkův Brod, Czech Republic; 4 Department of Clinical Biochemistry and Laboratory Diagnostics, 1st Faculty of Medicine, Charles University, General University Hospital, Prague, Czech Republic

**Keywords:** thyroid nodules, thyroid cancer, pregnancy, ultrasound, infertility

## Abstract

**Objective:**

Thyroid nodules are a common finding in the general population. The primary aim of the study was to determine the prevalence of thyroid nodules and cancer found by ultrasound (US) in women who underwent screening for thyroid dysfunction during pregnancy.

**Design:**

A double-centric, retrospective, cohort study.

**Patients and methods:**

We searched through medical records, including thyroid ultrasonography, of pregnant women who were positively screened for thyroid disorders (using thyroid-stimulating hormone and thyroid antibodies) from an unselected population (‘universal screening group’, *n*  = 690) and of women who underwent the testing based on the presence of clinical risk factors defined by American Thyroid Association (’case-finding group’, *n*  = 249).

**Results:**

Prevalence of benign and malignant thyroid nodules was lower in the ‘universal screening group’ than in the ‘case-finding group’ (9.9% vs 17.7%, *P*= 0.002, and 0.9% vs 7.2%, *P*< 0.001, respectively). Consistently, the thyroid cancer rate was lower among the nodules in the ‘universal screening group’ than in the ‘case-finding group’ (8.1% vs 29.0%, *P*= 0.003). Ultrasound EU-TIRADS (European Thyroid Imaging and Reporting Data System) category ≥4 had a 95.8% sensitivity for thyroid cancer. In palpable nodules, the prevalence of cancer was significantly higher than in the non-palpable ones (44.0% vs 2.2%, *P* < 0.001). In a multivariate regression analysis, thyroid nodules were associated with a history of infertility and parity.

**Conclusions:**

Compared to the data from cancer registries, universal screening allowed detecting thyroid cancer in pregnancy three to five times more frequently, but the cancer rate among nodules (8.1%) did not differ from the common population. US had very good sensitivity for thyroid cancer in pregnancy.

## Introduction

While the prevalence of thyroid nodules during pregnancy in areas with mild to moderate iodine deficiency varies between 3 and 21% (1, 2) and increases with increasing parity (3), data from areas with sufficient iodine supply are not available. After breast cancer, thyroid cancer is the second most common malignancy diagnosed during pregnancy (4). Approximately, 10% of thyroid cancers found during childbearing age occur during pregnancy or within the 12 months postpartum (5). The cancer rate of thyroid nodules diagnosed in pregnancy has been reported between 12 and 43% (6, 7, 8); however, the studies are limited by selection bias. The only study with an unselected population (*n*= 222) found a 15.3% rate of thyroid nodules and a 0% rate of cancer (2). Moreover, recent studies suggested an association of thyroid cancer with infertility (9, 10, 11), although some of them provided inconclusive results. Similarly, an association of thyroid cancer with diabetes mellitus has been reported (12), but data on gestational diabetes mellitus (GDM) are not available.

The aims of the study were (i) to compare the prevalence of thyroid nodules and cancer in women who underwent two different screening strategies for thyroid dysfunction in pregnancy: ‘universal screening’ and ‘case-finding strategy’ based on the presence of clinical risk factors defined by the American Thyroid Association (ATA) (13); (ii) to evaluate the outcome of cancer cases during follow-up; (iii) to analyse a diagnostic performance of ultrasound (US) for thyroid cancer in pregnancy; and (iv) to evaluate associations between thyroid nodules as well as cancers diagnosed during pregnancy and the history of thyroid diseases, reproductive factors, and GDM.

## Subjects and methods

### Subjects

**Figure 1:**
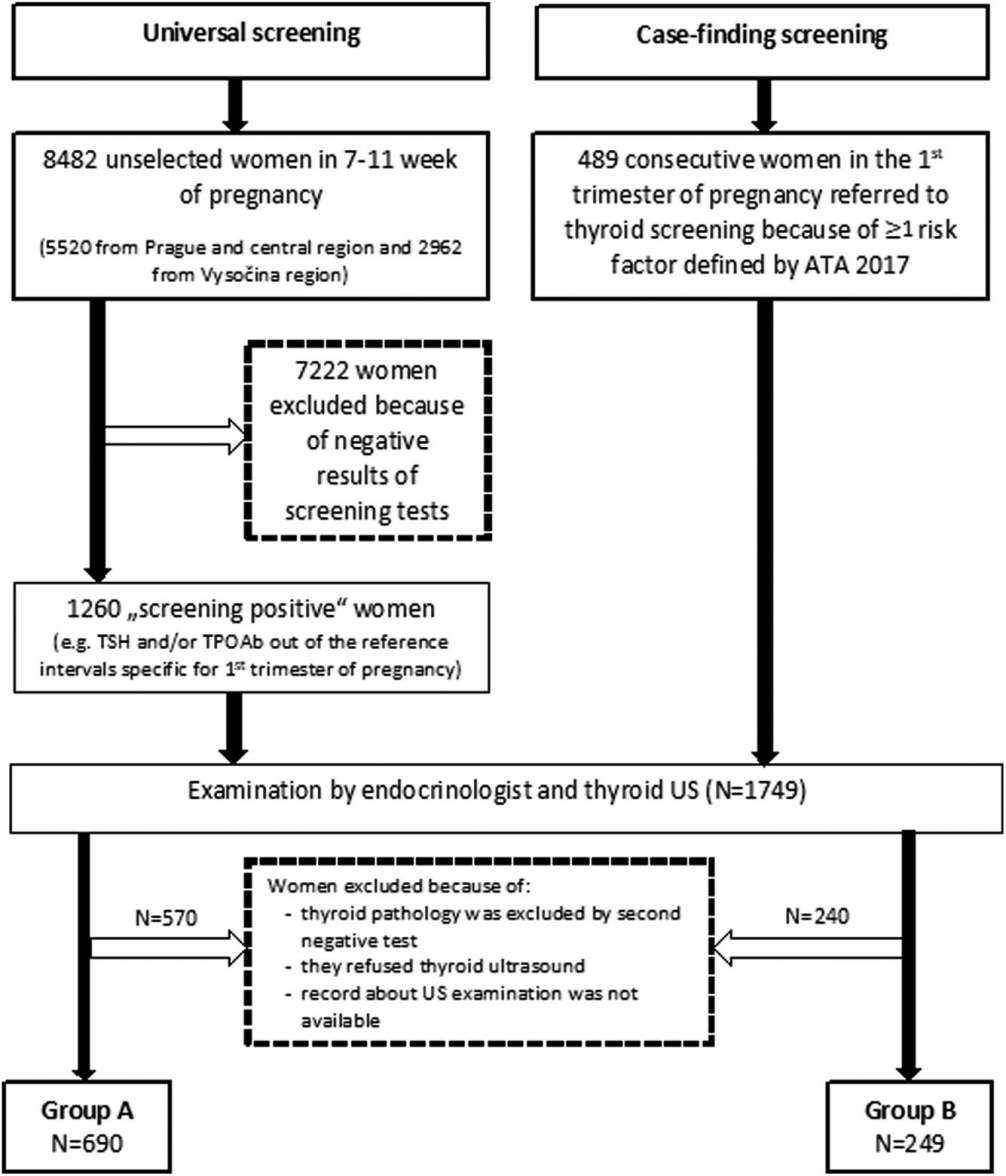
Flowchart of the study population. ATA, American Thyroid Association; TPOAb, antibodies to thyroid peroxidase; TSH, thyroid-stimulating hormone; US, ultrasound.

The study population of ‘universal screening’ (Group A) was recruited from an unselected cohort of women who underwent universal screening for thyroid diseases in pregnancy from January 2004 to December 2009 in two iodine-sufficient areas of the Czech Republic – Prague (*n* = 5520) and Vysočina region (*n* = 2962). In total, 8482 women were examined for TSH (thyroid-stimulating hormone) and TPOAb (antibodies to thyroid peroxidase) in weeks 9–11 of pregnancy. In total, 1260 of them (14.9%) were ‘positive’ defined as TSH and/or TPOAb out of the reference intervals specific for the first trimester of pregnancy. In order to demonstrate a selection bias of the previously reported unusually high incidence of thyroid cancer in pregnancy, we formed the ‘case-finding screening’ cohort consisting of women referred to thyroid biochemical screening based on the presence of at least one risk factor defined by the ATA: a history of hypothyroidism/hyperthyroidism or current symptoms/signs of thyroid dysfunction, known thyroid antibody positivity or presence of a goitre, history of head or neck radiation or prior thyroid surgery, age >30 years, type 1 diabetes mellitus (DM) or other autoimmune disorders, history of pregnancy loss, preterm delivery, or infertility, multiple prior pregnancies, family history of autoimmune thyroid disease or thyroid dysfunction, morbid obesity (BMI ≥40 kg/m^2^), use of amiodarone or lithium, or recent administration of iodinated radiologic contrast or residing in an area of known moderate to severe iodine insufficiency (13). In both original cohorts (‘universal screening’ and ‘case-finding screening’), all women with positive serum tests were referred to a thyroid US during pregnancy. Finally, 939 women (690 with a positive ‘universal screening’ – Group A and 249 with a positive ‘case-finding screening’ – Group B) with available records of thyroid US (Fig. 1).

In order to evaluate an association of thyroid cancer with thyroid and reproductive history and GDM, we retrospectively reviewed data from medical records. Finally, 397 women were available for this sub-analysis and 542 were excluded due to incomplete data. Infertility was defined by a medical record of (i) infertility in history, and/or (ii) treatment with fertility drugs, and/or (iii) any assisted reproduction technique used. GDM was defined as a medical record about its presence during a previous and/or current pregnancy.

### Thyroid ultrasound and fine-needle aspiration cytology

In women included in the study, one investigator retrospectively reviewed medical records about a thyroid US during pregnancy. Any spherical or ellipsoid lesion of size ≥5 mm at least in one dimension was defined as a thyroid nodule and a European Thyroid Imaging and Reporting Data System (EU-TIRADS) category (14) for each nodule was determined. Finally, 136 nodules were available for this sub-analysis.

Generally, the nodules of size ≥1 cm at least in one dimension and/or with high US suspicion (at least two US features of malignancy) were examined by fine-needle aspiration cytology (FNAC) and the others were followed by US. As thyroid cancers were defined the nodules with results of Bethesda category III, IV, V, or VI which underwent surgery and were confirmed by definitive histology. All women with cancer were followed regularly with a median follow-up of 11.3 years (minimum 9.3 and maximum 15.5 years).

As benign were defined (i) the nodules with FNAC results of Bethesda category II and no progression in size or nodule character during at least 5 years of US follow-up; (ii) the nodules with any FNAC result with benign definitive histology; (iii) the nodules ≥1 cm with FNAC results of Bethesda category I or III without surgery, but no progression in size or nodule character during at least 5 years of US follow-up; (iv) the nodules of size <1 cm without FNAC with no progression in size or nodule character during at least 5 years of US follow-up.

### Statistical analysis

The differences between proportions of nodules and cancers in different groups were evaluated by the chi-square test and the Fisher’s exact test, and relative risk (RR) was calculated. As we tested multiple hypotheses, Bonferroni correction (α/m) was used, where ‘α’ is the desired overall alpha level (0.05) and ‘m’ is the number of hypotheses/variables (*n* = 9). Statistically significant differences after Bonferroni correction are marked in bold. The differences in nodule size and serum TSH were assessed by the Mann–Whitney test. Sensitivity, specificity, positive predictive value (PPV), negative predictive value (NPV), and diagnostic accuracy of Bethesda category ≥III and EU-TIRADS category ≥4 were calculated and compared by the chi-square test.

As there were not enough cancer cases for a reliable multivariate logistic regression model, we analysed benign and malignant thyroid nodules together as dependent variables (’thyroid nodules’), and the associations with the following independent variables were tested: age, BMI, history of thyroid dysfunction, family history of thyroid diseases, history of infertility, history of abortions or preterm deliveries, history of use of oral contraceptives, parity, and history of GDM. A history of autoimmunity (including type 1 DM) was excluded from the analysis due to a low number of cases (*n*= 8).

All reported *P*-values are two-sided. Statistical software Sigmastat (Jandel Scientiﬁc, San Rafael, CA, USA) was used.

## Results

### Prevalence of thyroid nodules and cancers in women who underwent different screening strategies

The prevalence of benign and malignant thyroid nodules was considerably lower in women with positive universal thyroid screening (Group A) than in women from the ‘case-finding strategy’ (Group B) (Table 1). The difference was clearly more prominent in malignant nodules, resulting in a significantly lower overall cancer rate among the nodules in Group A than in Group B (6/74 (8.1%) vs 18/62 (29.0%), *P*= 0.003)).

**Table 1:** Prevalence of thyroid nodules and cancers in women positive in thyroid screening in pregnancy. *P*-value: level of significance (chi-square test).

	Group A (universal screening), *n* (%)	Group B (case-finding screening strategy), *n* (%)	*P*-value
Malignant nodules	6 (0.9)	18 (7.2)	<0.001
Benign nodules	68 (9.9)	44 (17.7)	0.002
No nodule	616 (89.2)	187 (75.1)	<0.001
Number of patients	690 (100)	249 (100)	

Of the 24 thyroid cancers, 19 were papillary (PTC), three were follicular variants of papillary thyroid cancer (FvPTC), one was a mix of PTC and FvPTC and one was follicular thyroid cancer (FTC). Four cancers were papillary thyroid microcarcinomas (PTMC) (≤1 cm) and four were multifocal (three MPTC and one >1 cm). Initially, there were four cancers of stage T1aN0M0, ten of T1bN0M0, two of T1bN1aM0, one of T2N0M0, one of T1aN1aM0, one of T1aN1bM0, four of T3N0M0, and one of T4N1bM1.

### Ultrasound characteristics, FNAC, and biochemical parameters in benign and malignant thyroid nodules diagnosed in pregnancy

The results are summarised in Table 2. All malignant nodules were confirmed histologically. Among the benign nodules, 9 were confirmed histologically after surgery (3 nodules of Bethesda category III and 6 of Bethesda category IV), 47 were of Bethesda category II and had no progression during at least 5 years of US follow-up, and 45 nodules were of Bethesda category I (*n* = 6), III (*n*= 5), or had no FNAC (*n*= 34), but had no progression in size and nodule character during at least 5 years of US follow-up.

**Table 2:** Comparison of ultrasound characteristics, FNAC results, and biochemical parameters in benign and malignant thyroid nodules found in pregnancy.

	Benign nodules	Malignant nodules	*P*-value
*n*	112	24	
Age of women^a^	30 (27.0–33.0)	30.5 (24.0–33.0)	0.968
US characteristics			
Size^a^	9.0 (6.0–12.0)	15.5 (12.0–20.0)	<0.001
>1 cm^b^	30 (26.8%)	19 (79.7%)	0.001
Multiple nodules^b^	30 (26.8%)	9 (37.5%)	0.421
EU-TIRADS 2^b^	21 (18.8%)	0 (0%)	0.046
EU-TIRADS 3^b^	32 (28.6%)	1 (4.2%)	0.023
EU-TIRADS 4^b^	54 (48.2%)	6 (25.0%)	0.064
EU-TIRADS 5^b^	5 (4.5%)	17 (70.8%)	<0.001
Biochemical parameters			
TSH^a^	1.8 (0.49–3.1)	1.78 (1.27–2.14)	0.746
Positive TPOAb^b^	46 (41.1%)	7 (29.2%)	0.393
FNAC (*n*= 91)			
Number of FNAC	67	24	
Bethesda I^b^	6 (9.0%)	0 (0%)	0.335
Bethesda II^b^	47 (70.2%)	2 (8.3%)	<0.001
Bethesda III^b^	8 (11.9%)	1 (4.2%)	0.436
Bethesda IV^b^	6 (9.0%)	2 (8.3%)	1.000
Bethesda V–VI^b^	0 (0%)	19 (79.2%)	<0.001

^a^Median (25–75%); ^b^*n* (%).

EU-TIRADS, European Thyroid Imaging and Reporting Data System; FNAC, fine-needle aspiration cytology; *n*, number of patients; *P*-value, level of significance (chi-square test, Fisher’s exact test; Mann–Whitney test); TPOAb, antibodies to thyroid peroxidase; TSH, thyroid-stimulating hormone; US, ultrasound.

Maximum size and the rate of nodules >1 cm were significantly higher in malignant as compared to benign nodules, whereas the rate of multiple nodules did not significantly differ. The distribution of EU-TIRADS categories was significantly different between benign and malignant thyroid nodules. Among 24 malignant nodules, 23 were of EU-TIRADS category 4 or 5, 1 was of EU-TIRADS category 3, and no nodule was of TIRADS category 2. The only malignant nodule in EU-TIRADS category 3 was histologically confirmed as a follicular variant of papillary thyroid cancer (FvPTC) with a maximum size of 47 mm (Table 2).

Among 24 malignant nodules, 2 were of Bethesda category II. The two cancers with false-negative initial FNAC were referred to surgery after delivery based on the progression during pregnancy. The first one was a nodule with a maximum size of 16 mm, retrospectively classified as EU-TIRADS category 5 and histologically confirmed as PTC of stage T1b. The second was one of the multiple nodules retrospectively classified as EU-TIRADS category 4 and histologically confirmed as multilocular PTC with a maximum size of 10 mm (T1a).

Sensitivity, specificity, NPV, and PPV of FNAC and EU-TIRADS classification for the prediction of thyroid malignancy are summarised in Table 3. Interestingly, the EU-TIRADS category ≥4 had very good sensitivity and NPV for detection of malignancy. The sensitivity and NPV of EU-TIRADS category ≥4 were at least equivalent to FNAC (Bethesda category ≥III), although specificity, PPV, and diagnostic accuracy were lower.

**Table 3:** Diagnostic performance of FNAC as compared to EU-TIRADS for thyroid malignancy in nodules diagnosed in pregnancy (*n*= 136).

	Sensitivity	Specificity	PPV	NPV	Accuracy
Bethesda category ≥III	91.7%	77.1%	61.1%	95.9%	81.2%
EU-TIRADS category ≥4	95.8%	47.3%	28.1%	98.2%	55.9%
*P*-value	0.843	<0.001	0.001	0.932	<0.001

FNAC: fine-needle aspiration cytology, EU-TIRADS: European Thyroid Imaging and Reporting Data System, PPV: positive predictive value (NPV), NPV: negative predictive value, *P*-value: level of significance (chi-suare test).

The median serum concentration of TSH and the prevalence of positive TPOAb did not significantly differ between women with benign and malignant nodules (Table 2).

### Management, complications, and outcomes of the cases with thyroid cancer

All women with cancer underwent total thyroidectomy, 8 (33.3%) during pregnancy and 16 (66.7%) after delivery. In 2 of 24 cases, complications during surgery occurred (1 transient hypoparathyroidism and 1 transient recurrent nerve paresis). Based on the ATA 2015 classification (15), 21 cases of cancer diagnosed in our study were initially classified as low risk, 2 as intermediate risk, and 1 as a high-risk tumour. Twenty women (83.3%) were treated by additional therapy with ^131^I radioiodine after pregnancy and 22 (91.7%) with levothyroxine suppressive therapy. All women with cancer were followed regularly with a median follow-up of 11.3 years (minimum 9.3 and maximum 15.5 years). We have found no adverse effect of ^131^I radioiodine treatment.

There was one case (4.2%) of biochemical (rising serum thyroglobulin) cancer persistence/recurrence in a woman with cancer initially classified as intermediate risk (T1bN1bM0). She was treated with two additional ^131^I doses (cumulative activity 21.5 GBq) and remains in incomplete biochemical remission 164 months after initial surgery.

On the contrary, the case initially classified as high risk (T4N1bM1) with lung metastases on the post-therapeutic scintigraphy was treated with an additional 5.5 GBq of ^131^I (cumulative dose 9.2 GBq) and is in complete remission 91 months after initial surgery.

### Sub-analysis of association of thyroid and reproductive history and GDM with thyroid nodules and cancer

The results are summarised in Tables 4 and 5. Notably, we found a significantly increased prevalence of thyroid cancer in women with a palpable nodule/goitre as compared to women with a negative neck palpation ((11/25 (44.0%) vs 8/372 (2.2%), *P*< 0.001)). Consistently, there was a significantly increased cancer rate among nodules in women with palpable nodules as compared to those with a negative neck palpation (RR 4.5, *P*< 0.001).

**Table 4:** Association of benign and malignant thyroid nodules found in pregnancy with history of thyroid diseases, autoimmunity, gestational diabetes mellitus, and reproductive factors (*n* = 397).

		Benign nodules			Malignant nodules		
	*n*	Proportion, *n* (%)	RR	*P*-value	Proportion, *n* (%)	RR	*P*-value
History of thyroid dysfunction							
Yes	75	9/75 (12.0)	NS	0.190	2/75 (2.7)	NS	0.513
No	322	62/322 (19.3)			17/322 (5.3)		
**Palpable goiter or nodule**							
Yes	25	10/25 (40.0)	2.4	0.007	**11/25 (44.0)**	**20.5**	<0.001
No	372	61/372 (16.4)			**8/372 (2.2)**		
Family history of thyroid diseases							
Yes	51	5/51 (9.80)	NS	0.156	2/51 (3.9)	NS	0.967
No	346	66/346 (19.1)			17/346 (4.2)		
History of autoimmunity (including type 1 DM)							
Yes	8	0/8 (0)	NS	0.386	0/8 (0)	NS	0.845
No	389	71/389 (18.3)			19/389 (4.9)		
History of GDM							
Yes	23	5/23 (21.7)	NS	0.828	4/23 (17.4 )	4.3	0.016
No	374	66/374 (17.6)			15/374 (4.0)		
History of infertility							
Yes	38	12/38 (31.6)	1.9	0.036	4/38 (10.5)	NS	0.179
No	359	59/359 (16.4)			15/359 (4.2)		
History of abortions or preterm deliveries							
Yes	84	16/84 (19.1)	NS	0.878	4/84 (4.7)	NS	0.782
No	313	55/313 (17.6)			15/313 (4.8)		
History of contraceptives use							
Yes	141	21/141 (14.9)	NS	0.309	5/141 (3.6)	NS	0.540
No	256	50/256 (19.5)			14/256 (5.5)		
Parous women							
Yes	149	35/149 (23.5)	1.6	0.034	9/149 (6.0)	NS	0.506
No	248	36/248 (14.5)			10/248 (4.0)		

*P*-value, level of significance (chi-square test and Fisher’s exact test), statistically significant differences after Bonferroni correction (α/m) where ‘α’ is the desired overall alpha level (0.05) and ‘m’ the number of hypotheses/variables (*n* = 9) are in bold.

DM, diabetes mellitus; GDM, gestational diabetes mellitus; NS, not significant;RR, relative risk.

**Table 5:** Independent predictors of presence of thyroid nodules (i.e. malignant and benign nodules together) in pregnancy in multivariate logistic regression analysis (*n* = 397).

	Odds ratio (95% CI)	*P*-value
Age	0.948 (0.895–1.003)	0.064
BMI	0.971 (0.918–1.028)	0.315
History of thyroid dysfunction	0.731 (0.365–1.466)	0.378
Family history of thyroid diseases	0.555 (0.244–1.262)	0.160
History of autoimmunity (including type 1 DM)^a^	–	–
History of GDM	2.340 (0.881–6.220)	0.088
**History of infertility**	**3.343 (1.540–7.255)**	**0.002**
History of abortions or premature deliveries	0.958 (0.508–1.807)	0.894
History of contraceptives use	0.686 (0.387–1.214)	0.196
**Parity**	**2.446 (1.408–4.249)**	**0.002**

^a^Excluded from the analysis due to low number of cases (*n* = 8).

Statistically significant associations are in bold.

DM, diabetes mellitus; GDM, gestational diabetes mellitus.

When we analysed the reproductive factors, we found a trend towards an increased prevalence of benign thyroid nodules in women with a history of infertility as compared to those without infertility (12/38 (31.6%) vs 59/359 (16.4%), *P*= 0.036) and in parous women as compared to nulliparous women (35/149 (23.5%) vs 36/248 (14.5%), *P*= 0.034)). However, the differences were not significant after the Bonferroni correction (Table 4). Consistently, the history of infertility and parity were associated with a presence of thyroid nodules regardless of their biological nature (i.e. malignant and benign together) in a multivariate logistic regression analysis (odds ratio 3.434 and 2.446, respectively, 95% CI 1.540–7.255 and 1.408–4.249, respectively, *P*= 0.002) (Table 5). Moreover, women with a history of infertility had a significantly higher rate of multiple nodules as compared to those with spontaneous conceptions ((6/38 (15.8%) vs 20/359 (5.6%), *P*= 0.038).

Although we found a slight trend towards a higher prevalence of thyroid cancer in women with previous/current GDM as compared to women with normal glucose metabolism during pregnancy ((4/23 (17.4%) vs 15/374 (4.0%), *P*= 0.016), the difference was not significant after the Bonferroni correction (Table 4). Consistently, no association of GDM with thyroid nodules was found in the multivariate regression model.

History of thyroid dysfunction, family history of thyroid diseases, and history of other autoimmunity were not significantly associated with thyroid nodules and cancers.

## Discussion

The incidence of thyroid cancer is increasing over time. Whether the increase is ‘true’ or inflated by ‘overdiagnosis’ has been debated (16, 17). Some authors attribute the increase to better medical care and the higher utilisation of imaging methods, that is US. This may be relevant to pregnant women who usually consume medical care more often and, therefore, thyroid nodules can be found more frequently. However, there is also a legitimate pathophysiological background of the association of thyroid nodules and cancer with pregnancy, for example, stimulation of thyroid cells by human chorionic gonadotropin (18), gonadotropins, gonadoliberins, and oestrogens, increased thyroid vascularity in pregnancy, or decreased immune surveillance of cancer (19).

In the first part of our study, we found the overall prevalence of thyroid nodules and cancers in an unselected cohort of women positive in universal thyroid screening in pregnancy (Group A) 74/690 (10.7%) and 6/690 (0.9%), respectively. Unsurprisingly, the prevalence of nodules in our population, recruited from iodine-sufficient areas, was lower than that reported for an iodine-deficient area (15.3%) (2). In our study, the overall cancer rate (8.1%) in nodules diagnosed during pregnancy was lower than reported in previous studies (12–43%) (6, 7, 8), and was similar to the common non-pregnant population (5–10%) (15). When we relate the 6 cases of thyroid cancer revealed in our study to the initial cohort of 8482 pregnant women with universal thyroid screening, we may approximate the prevalence of thyroid cancer in our cohort of unselected pregnant women to ca. 0.07%. This prevalence was 3.2-fold higher than in the Apulia National electronic database (22 cancer cases per 100,000 births) (20) and 4.9-fold higher than in the California Cancer registry (14.4 cancer cases per 100,000 births) (4).

In the group of women who underwent the ‘case-finding screening strategy’ (Group B) we found the prevalence of thyroid nodules and cancers 24.9 and 7.2%, respectively, which was significantly higher than in Group A (’universal screening’). When we related the 18 cases of thyroid cancer to the initial cohort of 489 pregnant women who underwent the ‘case-finding screening strategy’, we got a 3.7% prevalence of thyroid cancer in this group, that is much higher than in cancer registries and databases. Moreover, the cancer risk among the nodules in the ‘case-finding’ group was markedly higher (29.0%) than in common populations (5–10%) and our ‘universal screening’ group (8.1%), suggesting a selection bias.

In the sub-group of 136 women with revealed nodules, we compared diagnostic performances of thyroid US and FNAC for thyroid malignancy. The sensitivity and NPV of thyroid US (i.e. EU-TIRADS category ≥4) for thyroid cancer during pregnancy were similar to FNAC in our study. Importantly, there was no malignant nodule of EU-TIRADS category 2. The only malignant nodule of EU-TIRADS category 3 was histologically confirmed as a FvPTC, maximum size of 47 mm, which cannot be reliably diagnosed by FNAC anyway. Therefore, in our opinion, FNAC may be unnecessary in pregnant women with nodules of EU-TIRADS categories 2 and 3.

Furthermore, we retrospectively analysed the outcomes of 24 women with thyroid cancer found in pregnancy during follow-up. We found that most cases were referred to surgery after delivery (62.7% vs 37.3%), which is consistent with the current recommendation (13). Despite a relatively high overall rate of complications during surgery (8.3%), no complication was permanent. Although only one case of cancer was initially classified as high risk, 20 women (83.3%) were additionally treated with ^131^I radioiodine and 22 (91.7%) with levothyroxine suppressive therapy. It is in contradiction with recent ATA 2015 guidelines (15) and indicates a significant over-treatment from today's perspective. This approach could result in unnecessary anxiety of patients who are new parents. However, such aggressive management of thyroid cancer diagnosed in pregnancy was not rare at the time of the study (2004–2009) in our country. The reason could be mainly a disputable concern about cancer progression in young women after delivery. Generally, women with thyroid cancer found in pregnancy had excellent outcomes in our study (only one case of biochemical, but not structural disease persistence/recurrence). Although in our retrospective study we cannot support it with exact data, we believe that it is rather due to low aggressiveness of the cancers than due to the aggressive treatment.

In the sub-group of 397 women, we analysed an association of thyroid nodules and cancer with thyroid history, reproductive factors, and GDM. We found a markedly higher prevalence of thyroid cancer in palpable nodules as compared to non-palpable ones (RR 20.5). This is in contradiction with the common population where a similar risk of cancer in non-palpable and palpable nodules has been reported (21).

Despite large experimental evidence supporting an association of several reproductive factors with thyroid cancer, clinical studies reported controversial results (3, 22, 23, 24, 25, 26). In our study, we found no significantly increased prevalence of thyroid nodules and cancer in infertile women, parous women, and women with a history of use of oral contraceptives in univariate analysis. This is partially conclusive with the recent meta-analysis by Yu *et al.* reporting only a slight risk of thyroid cancer in women treated with infertility drugs (27) and conclusive with many other studies that reported negative results (11, 28, 29, 30, 31, 32, 33). However, thyroid nodules regardless of their biological nature (i.e. benign and malignant together) were independently associated with infertility and parity in our multivariate regression model.

To our knowledge, our study is the first one evaluating an association between thyroid cancer and GDM. Generally, diabetic patients have a higher risk of various types of cancer and, also, the association with thyroid cancer was reported (12, 34). Although we found a slight trend of increased prevalence of thyroid cancer in pregnant women with a history of GDM as compared to those with normal glucose metabolism (17.4% vs 4.0%), the difference was not significant, and no association was found in the multivariate regression model.

Our study has several limitations. First, due to its retrospective character, we had a high number of excluded patients, mainly because of a lack of available medical records, including thyroid US. However, all women in both cohorts with positive serum tests were referred to thyroid US regardless of other clinical factors, and also the rate of lacking US records was similar in both groups (45% in Group A and 49% in Group B). Therefore, in our opinion, a selection bias potentially overestimating the true prevalence of thyroid nodules and cancer in Group A recruited from the ‘universal screening’ cohort is rather limited. Further, we analysed thyroid US only in the women with positive screening. As increased TSH is positively associated with thyroid nodules (35), the true prevalence of unrecognised nodules and cancers may be somewhat lower in unselected pregnant women. Furthermore, due to the population-based character of our study, we had quite a low number of cancers for analysis of diagnostic performance of EU-TIRADS risk stratification and outcomes and we also cannot separately evaluate histological subtypes of cancer. Finally, a selection bias may have skewed the results of the association sub-analysis because some of the independent variables are among the clinical risk factors defined by ATA.

In conclusion, although the ‘universal screening’ (i.e. general testing of serum TSH and TPOAb) increased the observed thyroid cancer incidence in pregnancy approximately three- to five-fold as compared to the data from cancer registries, the cancer rate among the nodules did not differ from the common population. On the contrary, an unusually high cancer rate (29.0%) among the nodules in the ‘case-finding’ group (i.e. testing of serum TSH and TPOAb only in women with positive clinical risk factors) probably indicates a selection bias, because the presence/history of thyroid nodule/goitre is a clinical risk factor. As thyroid cancer was much more prevalent in palpable nodules than in non-palpable nodules, clinicians should pay special attention to thyroid palpation in pregnant women. As the US had good sensitivity for thyroid cancer during pregnancy, FNAC may be unnecessary in pregnant women with nodules of EU-TIRADS categories 2 and 3. Although infertility and parity were associated with thyroid nodules in our multivariate regression model and a history of oestrogen stimulation may be a plausible explanation, further studies are needed to clarify these associations.

## Declaration of interest

The authors declare that there is no conflict of interest that could be perceived as prejudicing the impartiality of the research reported.

## Funding

This research was funded by the project MH CZ - DRO (General University Hospital in Prague - VFN, 00064165).

## Ethical statement

The study complies with the guidelines for human studies and the research was conducted ethically in accordance with the World Medical Association Declaration of Helsinki. All participants signed informed consent and the protocol of the screening programme was approved by the Ethics Committee of Generally University Hospital and the Ethics Committee of the University of Hradec Králové. This retrospective study was approved by the Ethics Committee of Generally University Hospital in Prague.

## Author contribution statement

Jan Jiskra, Jiří Horáček, Sylvie Špitálníková, Jan Paleček, Zdeňka Límanová, Jan Krátký, Drahomíra Springer, Kristýna Žabková and Hana Vítková substantially contributed to the study.
